# Comparative Analysis of Five Observational Audit Tools to Assess the Physical Environment of Parks for Physical Activity, 2016

**DOI:** 10.5888/pcd13.160176

**Published:** 2016-12-15

**Authors:** Rodney P. Joseph, Jay E. Maddock

**Affiliations:** 1 College of Nursing and Health Innovation, Arizona State University, Phoenix, Arizona; 2 Department of Environmental and Occupational Health, School of Public Health, Texas A&M Health Science Center, College Station, Texas.

## Abstract

We reviewed prominent audit tools used to assess the physical environment of parks and their potential to promote physical activity. To accomplish this, we manually searched the Active Living Research website (http://www.activelivingresearch.com) for published observational audit tools that evaluate the physical environment of parks, and we reviewed park audit tools used in studies included in a systematic review of observational park-based physical activity studies. We identified 5 observational audit tools for review: Bedimo-Rung Assessment Tool–Direct Observation (BRAT-DO), Community Park Audit Tool (CPAT), Environmental Assessment of Public Recreation Spaces (EAPRS) tool, Physical Activity Resource Assessment (PARA), and Quality of Public Open Space Tool (POST). All 5 tools have established inter-rater reliability estimates ranging from moderate to good. However, BRAT-DO is the only tool with published validity. We found substantial heterogeneity among the 5 in length, format, intended users, and specific items assessed. Researchers, practitioners, or community coalition members should review the goal of their specific project and match their goal with the most appropriate tool and the people who will be using it.

## Introduction

The relationship between physical activity and health is well established (1–3); however, despite the benefits of physical activity, most Americans are insufficiently active (4). Parks have the potential to influence physical activity behaviors (5) because they are normally close to residential neighborhoods, have specific structures designed for physical activity, and usually can be accessed at no cost. However, because a park is present does not necessarily mean that it is supportive of physical activity. The degree to which parks encourage physical activity is largely determined by their design, their perceived safety, and the presence of park structures that support physical activity (6–9).

Researchers have developed various observational audit tools to examine the physical environment of parks and their potential to promote physical activity. However, the format, length (ie, number of items), assessment methods, literacy required to complete assessments, and characteristics and features of these tools vary widely. We reviewed 5 prominent audit tools for assessing the physical environment of parks to assist public health practitioners, researchers, and community members in determining which audit tool is best for them to use for a specific research project or evaluation.

## Basis for Comparison

We conducted a selective review of environmental audit tools. To identify relevant measures, we manually searched the Active Living Research website (http://www.activelivingresearch.com) for published observational audit tools that evaluate the physical environment of park-based settings, and we reviewed tools used by researchers in studies included in a systematic review of observational studies of park-based physical activity (10). When selecting tools for inclusion in the review, we focused on those that evaluated both the open spaces and physical structures of parks. Our initial search procedures revealed 7 potential tools for inclusion in the review: Bedimo-Rung Assessment Tool–Direct Observation (BRAT-DO) (11), Community Park Audit Tool (CPAT) (12), Environmental Assessment of Public Recreation Spaces (EAPRS) tool (13), Physical Activity Resource Assessment (PARA) instrument (14), Path Environment Audit Tool (PEAT) (15), Rural Active Living Assessment (RALA) Tools (16), and Quality of Public Open Space Tool (POST) (17). After reviewing the tools and applying our inclusion criteria, we selected 5 for inclusion in the review: BRAT-DO (11), CPAT (12), EAPRS (13), PARA (14), and POST (17). PEAT and RALA tools were excluded because they did not include a detailed assessment of the physical features of parks (PEAT focuses on trails or paths and RALA focuses on the physical features and policy characteristics of entire towns and communities rather than individual parks).

In comparing the measures, we first evaluated the following characteristics: length, intended users, data collection method, reliability, and validity. Next, we performed a more detailed analysis of each tool based on specific features assessed. We reviewed each measure and classified items in the following categories: activity areas, sitting and resting features, landscape features, facilities (rest rooms, showers, event venues), eating and drinking features, access and neighborhood characteristics, signage, safety-related features, incivilities (evidence of offensive behavior, eg, litter, graffiti, loud noise), and the park’s condition and maintenance. We placed items not fitting into these categories in a category called “other items assessed.” In comparing the measures, our first level of review was to evaluate the following characteristics: length, intended users, data collection method, reliability, and validity. The second level of review was a more detailed analysis of each tool based on specific features assessed. Last, we compared the 5 tools on the basis of the outcomes assessed in the first 2 levels of review.

## Overview of Audit Tools

### Community Park Audit Tool (CPAT)

CPAT (http://www.activelivingresearch.com) comprises 28 items and is divided into 4 sections: park information, access and surrounding neighborhood, park activity areas, and park quality and safety. CPAT was designed for community members, stakeholders, and researchers to easily assess community parks with an emphasis on the physical activity of youths. Inter-rater reliability of the CPAT is generally moderate to high; most items have agreement ranging from 80% to 90% (12). CPAT was originally developed as a pencil-and-paper instrument (6 pages), but an electronic version is now available.

### Bedimo-Rung Assessment Tool–Direct Observation (BRAT-DO)

BRAT-DO (http://publichealth.lsuhsc.edu/Faculty_Pages/rung/index_files/page0004.htm) is a pencil-and-paper tool designed to identify and evaluate the physical structures and characteristics of parks. BRAT-DO is intended for use by researchers, consists of 181 items (16 pages), and assesses the following domains: access, condition, aesthetics, features, and safety. The tool has established inter-rater reliability among people trained in the assessment protocol; inter-rater agreement across domains ranged from 67.6% to 100% (11). Validity was determined by comparing outcomes achieved by trained data collectors against experts. Average inter-rater agreement ranged from 68.3% to 88.3%. 

### Environmental Assessment of Public Recreation Spaces (EAPRS) Tool

EAPRS (13) (http://www.activelivingresearch.com) is the most comprehensive observational audit measure available. It is in its sixth revision and is available in pencil-and-paper format. EAPRS contains 751 items in 16 sections and is 59 pages long. Inter-rater reliability varies by section or domain assessed; however, most items in the tool demonstrate moderate-to-high reliability estimates (eg, κ ≥ .60) (13). 

### Physical Activity Resource Assessment (PARA) instrument

PARA (14) (http://grants.hhp.coe.uh.edu/undo/?page_id=21) is a brief, 1-page (49 items) instrument that assesses park type, features, amenities, qualities, and incivilities. It was originally developed to assess resources in low-income, urban communities that surround public housing developments (14). Use of PARA is not limited to assessment of parks or playgrounds and can be used to audit other physical environments. Inter-rater reliability is moderate to high (κ > .77). 

### Quality of Public Open Space Tool (POST)

POST (17) (http://www.see.uwa.edu.au/research/cbeh/projects/post) is a pencil-and-paper–based tool designed to evaluate physical attributes of public open spaces, including parks, that may influence physical activity. POST is 2.5 pages in length and consists of 88 items. Inter-rater agreement for the items ranges from 50% to 98%, with most items demonstrating greater than 85% agreement (18).

## Comparative Analysis

We compared the 5 tools by length, intended users, data collection method, reliability, and validity (Table 1). PARA was the shortest at 1 page (49 items), and EAPRS was the longest at 59 pages (751 items). The other 3 measures ranged in length from 2.5 to 16 pages. All 5 are designed for use by researchers and public health practitioners; CPAT and PARA are also appropriate for community members. All 5 measures are available in pencil-and-paper format. CPAT is also available in an electronic format for use on tablet or smartphone. The assessment protocols for all 5 measures are similar: parks are divided into segments or activity areas, and each segment is evaluated individually.

**Table 1:** Characteristics of 5 Audit Tools to Assess the Physical Environment of Parks for Physical Activity, 2016

Measure	Length	Intended Users	Data Collection Method	Reliability^a^	Validity^a^
CPAT (12)	6 pages, 28 items	Researchers and Community members	Pencil and paper, electronic versions for tablet and smartphone	Most items (ie, all but 4) have inter-rater reliability of κ ≥ .70	Not reported
BRAT- DO (11)	16 pages, 181 items	Researchers	Pencil and paper	Overall domain^b^ inter-rater reliability = .87; overall geographic area^c^ inter-rater reliability = .88.	Overall domain^b^ validity = .79; overall geographic area^c^ validity = .82
EAPRS (13)	59 pages, 751 items	Researchers	Pencil and paper	65.6% of items have good-to-high inter-rater reliability (κ ≥ .60)	Not reported
PARA (14)	1 page, 49 items	Researchers and community members	Pencil and paper	Overall inter-rater reliability for each item is κ > .77.	Not reported
POST (17)	2.5 pages, 88 items	Researchers	Pencil and paper	67% of items have good-to-high inter-rater reliability (κ ≥ .60)	Not reported

Abbreviations: BRAT-DO, Bedimo-Rung Assessment Tool–Direct Observation; CPAT, Community Park Audit Tool; EAPRS, Environmental Assessment of Public Recreation Spaces tool; PARA, Physical Activity Resource Assessment; POST, Quality of Public Open Space Audit Tool.

a Reliability and validity estimates are based on original publications of each audit tool reported by the authors of each tool. The heterogeneity of how reliability outcomes are presented reflects the heterogeneity of how these outcomes were reported. Kappa are percentages of agreement.

b Domain items assess the access, condition, aesthetics, features, and safety of parks.

c Geographic area items are street, court, green space, path, playground, and sports field.

All 5 measures have established reliability with moderate-to-good inter-rater reliability estimates (Table 1). BRAT-DO was the only measure with validity estimates determined by inter-rater agreement (ie, percentage agreement) between trained data collectors and expert data collectors (ie, data from expert data collectors were viewed as the gold standard or criterion). Overall domain validity for BRAT-DO (ie, for items assessing access, condition, aesthetics, features, and safety) was 78.7%, and overall geographic validity (ie, for items assessing streets, courts, green spaces, paths, playgrounds, and sports fields) was 81.5%.

## Park Features Assessed

A detailed table illustrating the items assessed by each audit tool, which illustrates how we derived the information presented in Table 2, is available from the authors.

**Table 2:** Features and Amenities Assessed by 5 Park Audit Tools to Assess the Physical Environment of Parks for Physical Activity, 2016

Park Amenity	CPAT (12)	BRAT-DO (11)	EAPRS (13)	PARA (14)	POST (17)
	No. of Items Assessing Each Area				
**Activity areas**					
Children play areas	1	6	8	1	7
Courts	3	2	4	3	3
Fields and open green spaces^a^	4	2	4	2	4
Golf courses	NA	1	3	NA	NA
Running and walking trails and paths	1	1	3	2	1
Water activity areas^b^	4	4	4	1	3
Other types of activity features	4	1	11	3	1
**Sitting and resting^c^**	2	2	4	1	2
**Landscaping **	5	5	9	3	6
**Facilities^d^**	2	3	3	3	4
**Eating and drinking **	2	3	4	1	3
**Park access and neighborhood characteristics**	10	8	6	1	4
**Signage**	8	7	3	2	1
**Safety-related features^e^**	4	2	4	1	1
**Incivilities^f^**	4	4	1	5	3
**Dogs**	2	NA	1	1	2

Abbreviations: BRAT-DO, Bedimo-Rung Assessment Tool–Direct Observation; CPAT, Community Park Audit Tool; EAPRS, Environmental Assessment of Public Recreation Spaces tool; NA, not applicable; PARA, Physical Activity Resource Assessment; POST, Quality of Public Open Space Audit Tool.

a Open grassy areas, soccer fields, baseball or softball fields, football fields, and cricket fields.

b Swimming and wading pools, splash pad, beach or river, ponds and lakes, streams and creeks.

c Benches, tables, seat walls, and bleachers.

d Equipment for rent, restrooms or toilets, showers, changing rooms, event venues or stages, and meeting rooms.

e Presence of telephones and emergency call boxes, park staff on site, lighting, and threatening persons or behavior.

f Offensive behavior (eg, litter, graffiti, loud noise).

**Children play areas*.*** All 5 tools include at least 1 item to assess the presence of play structures or play sets. BRAT-DO, EAPRS, and POST provide the most detailed assessment of children play structures, because they assess specific types of structures (eg, swings, slides, climbing equipment).

**Courts.** All 5 tools assess the presence of courts; however, the type of court varies. All 5 assess basketball and tennis courts. Three (CPAT, EAPRS, and PARA) also assess volleyball courts. Only EAPRS assesses the presence of handball courts, and only POST assesses netball courts.

**Fields and open green spaces*.*** All 5 measures evaluate the presence of soccer and softball or baseball fields. Other field types evaluated by measures were football or rugby (CPAT, BRAT-DO, and EAPRS) and cricket (POST). CPAT, BRAT-DO, and EAPRS also included items to assess the presence of open green spaces and fields for general play.

**Golf courses.** EAPRS and BRAT-DO are the only 2 measures that assess the presence of golf courses. The EAPRS was the most detailed, because it assessed the presence of regular (9- or 18-hole), miniature, and Frisbee golf courses. BRAT-DO evaluated the presence of a regular golf course.

**Running and walking features*.*** All 5 measures assess the presence of unpaved trails or paths for running and/or walking activities. Two measures, PARA and EAPRS, also evaluate the presence of sidewalks.

**Water features*.*** Four measures, CPAT, BRAT-DO, EAPRS, and POST, evaluate the presence of ponds and lakes and creeks and streams within parks. CPAT, BRAT-DO, EAPRS, and PARA also include items to evaluate the presence of swimming and wading pools. CPAT is the only measure assessing splash pads. Only BRAT-DO and EAPRS assess whether the park is located on a beach or riverfront.

**Other types of activity areas*.*** The audit tools reviewed also assess the presence of other activity areas. For example, CPAT and PARA assess the presence of exercise stations or exercise equipment. Likewise, CPAT and EAPRS assess the presence of dedicated dog parks. EAPRS provides the most comprehensive assessment, because it is the only measure to assess batting cages, BMX (bicycle motocross) tracks, driving ranges, horseshoe pits, shuffleboards, and other features.

**Sitting and resting features*.*** All 5 tools evaluate the presence of picnic tables. All except PARA assess the presence of benches. EAPRS also assesses the presence of other types of sitting or resting features, including seat walls and bleachers.

**Landscaping features*.*** All measures include items to assess landscape features. Common items assessed are trees, flower and garden areas, fountains, and areas with shade.

**Facilities*.*** All 5 measures include items that evaluate the presence of restrooms and trash cans. BRAT-DO was the only measure to assess if play or athletic equipment is available for rent. PARA was the only measure to assess the presence of showers or changing rooms, and POST was the only measure to evaluate the presence of meeting rooms. EAPRS and POST also assessed the presence of event venues or stages.

**Eating and drinking features.** All five tools assess the presence of water fountains. BRAT-DO, EAPRS, and POST also evaluate the presence of grills or barbeques. All measures except PARA assess the presence of other food sources (eg, vending machines, concession stands).

**Access and neighbor characteristics*.*** All measures include items to evaluate park access and environmental supports for transit to parks. Common items include the presence of sidewalks or paths connecting to the park, adjacent roadways and parking lots, and the presence of bike racks. CPAT was the only measure to assess presence of a nearby public transit stop. Only 2 measures, CPAT and BRAT-DO, assess whether a park can be locked or is locked at the time of audit. All measures except PARA assess the quality of the neighborhood surrounding the park.

**Signage*.*** CPAT and BRAT-DO are the most detailed measures of signage, because they evaluate the presence of posted park rules and regulations, hours, event programming, dog and pet user rules, and information on how to reserve areas and other types of signage. PARA and POST are the least detailed. EAPRS assesses the presence of signs for rules and regulations, maps, and event programming.

**Safety-related features*.*** All measures assessed lighting. CPAT, BRAT-DO, and EAPRS also assess the presence of telephones and emergency call boxes and onsite park staff. CPAT was the only measure to assess threatening behavior among park users (ie, presence of gangs and alcohol or drug use). PARA and POST are the least detailed in assessing park safety features; they assess only presence of lighting.

**Incivilities*.*** All measures assessed the presence of incivilities. CPAT, BRAT-DO, PARA, and POST are the most detailed. Example items assessed were litter, graffiti, and loud noise. EAPRS included only a few items to assess incivilities.

**Condition and maintenance.** All measures except POST evaluate the condition and maintenance of parks. However, the method in which the measures assess these aspects differs. CPAT, BRAT-DO, and EAPRS first assess the presence of a specific park feature or amenity and then use a separate additional item to assess its condition and maintenance. PARA uses a combined scale to assess both the presence and condition of the features.

**Dogs.** All measures except the BRAT-DO include items to assess the presence of dogs. CPAT and POST further expand on this topic by including an item to assess if waste litter bags for dogs are present.

## Considerations for Selecting an Audit Tool

Because of the heterogeneity of the 5 audit tools reviewed, practical guidance on the usefulness of a tool for specific research projects is warranted. Table 3 illustrates common audit scenarios faced by both researchers and public health practitioners and the corresponding audit tools best suited for each scenario.

**Table 3:** Potential Park Audit Scenarios and Corresponding Audit Tools Best Suited for Each Scenario to Assess the Physical Environment of Parks for Physical Activity, 2016

Park Audit Scenario	Suggested Audit Tool(s)	Commentary
Have community members perform park audits	CPAT, PARA	The CPAT and PARA are most advantageous when having lay community members perform park audits, as both require low literacy levels and can be completed in a short time frame. If the community comprises predominately low-income members, the PARA may be of particular interest, because it was originally developed for use in low-income communities. The EAPRS, POST, and BRAT-DO are not ideal for use by lay community members unless extensive training is provided.
Obtain a quick assessment of a park’s potential to promote physical activity	PARA, POST	The PARA was the shortest measure reviewed (ie, 1 page) and can be completed in less than 10 min for a medium-sized park (ie, less than 1 city block). The POST is a little longer in length than the PARA (ie, 2.5 pages vs 1 page) and includes a more detailed assessment of park features and amenities than the PARA. However, we estimate the amount of time to conduct a park audit using the POST is not much longer than an audit using the PARA.
Obtain a comprehensive assessment of a park’s features	EAPRS, BRAT-DO	The EAPRS is the most comprehensive measure and includes detailed assessment for almost any type of park feature that may be present. However, data collectors using this measure will need extensive training, and the time needed to assess each park will likely take substantially longer than assessments made with shorter audit measures. The BRAT-DO, while less detailed than the EAPRS, strikes a good balance between detail of assessment, level of training for data collectors, and time to complete the audit. Moreover, it has better overall reliability estimates than the EAPRS. The decision to use either the EAPRS or BRAT-DO for a specific project should be determined based on the ultimate goal of the park audit and available resources to train data collectors and perform the park assessments.
Assess a park’s potential to promote child or adolescent physical activity	CPAT	The CPAT is ideal to assess a park’s potential to encourage child or adolescent physical activity, because this measure was specifically developed with a focus on assessing play areas for both children and adolescents. The EAPRS and BRAT-DO are also useful tools to evaluate a park’s potential to promote physical activity among children and adolescents because of their detailed assessment of park features designed for children. However, these measures are considerably longer than the CPAT and require a higher level of skill and literacy. Moreover, the EAPRS has lower inter-rater reliability estimates than both the CPAT and BRAT-DO. Accordingly, unless an extensive level of detail is needed, we recommend the CPAT be used to assess a park’s potential to encourage child and adolescent physical activity.
Assess a park’s potential to promote physical activity among people of all ages	CPAT, BRAT-DO, EAPRS, PARA, POST	All 5 of the audit tools reviewed were designed to assess a park’s potential to promote physical activity among people of all ages (although the CPAT placed a greater emphasis on children and adolescents). When selecting an audit tool to use for a specific project, we encourage data collectors to consider other factors that may influence the type of assessment needed (eg, skill needed, length of tool, characteristics of data collectors).
Obtain a middle-of-the-road assessment when considering factors of time to complete the assessment, skill required, validity, reliability, and level of detail needed	CPAT, BRAT-DO	The CPAT and BRAT-DO are well-balanced measures that can be used by researchers, public health practitioners, and community members alike. These 2 measures have good reliability estimates, include a moderate-to-high level of detail, and can be completed in a reasonable amount of time. Therefore, we recommend both of the measures for researchers and practitioners looking to conduct a general assessment of a park’s potential to encourage physical activity.

Abbreviations: BRAT-DO, Bedimo-Rung Assessment Tool–Direct Observation (11); CPAT, Community Park Audit Tool (12); EAPRS, Environmental Assessment of Public Recreation Spaces tool (13); PARA, Physical Activity Resource Assessment (14); POST, Quality of Public Open Space Audit Tool (17).

## Discussion

Our comparative analysis showed heterogeneity in the length of the audit tools. Two tools, PARA and CPAT, were designed for researchers, public health practitioners, and community members, where BRAT-DO, EAPRS, and POST were designed for researchers and public health practitioners only. A positive finding of our review was that all 5 audit tools have inter-rater reliability estimates that range from moderate to good. This finding is encouraging and speaks to the organization and clarity of the tools and their training manuals. In reference to validity, BRAT-DO is the only tool to report validity outcomes. Studies are needed to establish the validity of the other audit tools.

Choice of an audit measure for a project should be based on various factors, which include the level of detail needed, education and skill level of the people performing the assessment, time needed to complete the assessment, target population of park users (ie, children vs adults), and resources available to train assessors and to conduct the assessment. When choosing a tool, researchers will often face a trade-off among these factors, because each tool will not perfectly address all project specifications. Despite this trade-off, all 5 measures reviewed show promise for evaluating the built environment of parks for the support of physical activity.

Although comprehensive, our review has limitations. We limited our selection to audit tools used in studies included in our systematic review of park-based physical activity studies (10) and those available on the Active Living Research website. Therefore, other audit tools may be available that were not included in this review. Additionally, the process of comparing the audit tools was challenging because of the heterogeneity of the format, detail, and length of the tools. Accordingly, our comparative analysis oversimplified many of the park features assessed by each audit tool. This was particularly the case with our review of EAPRS, because this tool collects extensive detail for every feature or aspect assessed. We also would have liked to compare time required to audit a park of similar size and features using each tool. However, we were unable to perform this comparison because of the heterogeneity of parks used in the development and validation of the audit tools. Studies are needed to compare the approximate amount of time it takes to audit a standardized park with each tool.

Despite these limitations, this review has several strengths. To our knowledge, this is the first review of observational audit tools to evaluate the physical environment of parks in relation to physical activity. Findings are designed to help researchers, public health practitioners, and community coalitions identify an appropriate tool for a particular project. Another strength was the high level of detail used to evaluate the 5 measures. We compared various aspects of each tool, including length, assessment method, intended users, reliability, validity, and specific park features assessed. This level of detail strengthens the quality and usefulness of the review.

Our review was intended to provide guidance in selecting the best audit tool for a proposed park evaluation project. When selecting an appropriate tool, researchers should review the goal of their project and match their goal with the most appropriate tool, because the method of assessment will have a direct relationship with validity and usefulness of the outcomes. Likewise, researchers may find it necessary to use multiple audit tools or to combine sections of different audit tools to evaluate the specific features of a park environment.
